# Case Report: Idiopathic Spinal Cord Herniation: An Overlooked and Frequently Misdiagnosed Entity

**DOI:** 10.3389/fsurg.2022.905038

**Published:** 2022-05-20

**Authors:** Chenlong Yang, Guozhong Lin, Jia Zhang, Jun Yang, Jingcheng Xie

**Affiliations:** Department of Neurosurgery, Peking University Third Hospital, Beijing, China

**Keywords:** idiopathic spinal cord herniation, diagnosis, surgery, outcome, case report

## Abstract

**Background:**

Idiopathic spinal cord herniation is an extremely rare entity that is characterized by protrusion of the spinal cord through a defect in the ventral dura. Due to the paucity of enough clinical evidence, the treatment and prognosis of idiopathic spinal cord herniation are still elusive. Herein, we reported a case of idiopathic spinal cord herniation occurring at the C7–T1 levels that was treated by surgical reduction.

**Case description:**

A 44-year-old Chinese woman presented with a 5-year history of numbness and weakness in the bilateral lower limbs. Spinal magnetic resonance imaging demonstrated ventral displacement of the spinal cord at the C7–T1 levels, and there seemed to be a cuneiform space-occupying lesion dorsal to the spinal cord. A diagnosis of the spinal intradural extramedullary tumor was suspected. An exploratory operation was performed via a posterior midline approach. Intraoperatively, we found a defect in the ventral dura through which the spinal cord herniated to the epidural space. After the herniated parenchyma was returned, an artificial dura matter was used to repair the defect. The postoperative course was uneventful. After a 3-month follow-up, the lower-extremity weakness was significantly improved, and there was no recurrence of the spinal cord herniation.

**Conclusion:**

Preoperative diagnosis of idiopathic spinal cord herniation is exceedingly challenging. Surgical reduction of the herniated spinal cord with the repair of the dural defect is an effective approach for the treatment of this rare disorder, and the surgical outcome is favorable.

## Introduction

Idiopathic spinal cord herniation is an extremely rare entity that is characterized by protrusion of the spinal cord through a defect in the ventral dura. This pathological condition was originally identified by Wortman et al. in 1974, in which case the herniated spinal cord was incidentally noticed during a thoracotomy operation for disc herniation (1). Till now, existing documents of idiopathic spinal cord herniation are only limited to case reports, and the preoperative diagnosis is exceedingly challenging (2–8). The pathogenesis of dural defect formation and spinal cord herniation remains unknown; over the past decades, several causes have been hypothesized based on the intraoperative observations, such as trauma (9), congenital deficiency in the dura (10), and pressure-related erosion secondary to the intervertebral disc herniation (11). Due to the paucity of enough clinical evidence, the treatment and prognosis of idiopathic spinal cord herniation are still elusive (12). Herein, we reported a case of idiopathic spinal cord herniation occurring at the C7–T1 levels that was treated by surgical reduction.

## Case Description

### History

A 44-year-old Chinese woman presented to us with a 5-year history of numbness and weakness in the bilateral lower limbs. Her symptoms had been aggravated during the last year prior to admission. The previous medical history was unremarkable, and the patient denied any back trauma. She was unable to walk steadily. There were no bladder or bowel disturbances.

### Physical Examination

Physical examination revealed a decreased muscle strength (Grade 4/5) in the bilateral lower extremities and attenuated pinprick and temperature sensation below the T1 dermatome. Her patellar tendon reflex and Achilles tendon reflex were hyperactive bilaterally, while pathological reflexes were not induced.

### Radiological Examination

**Figure 1:**
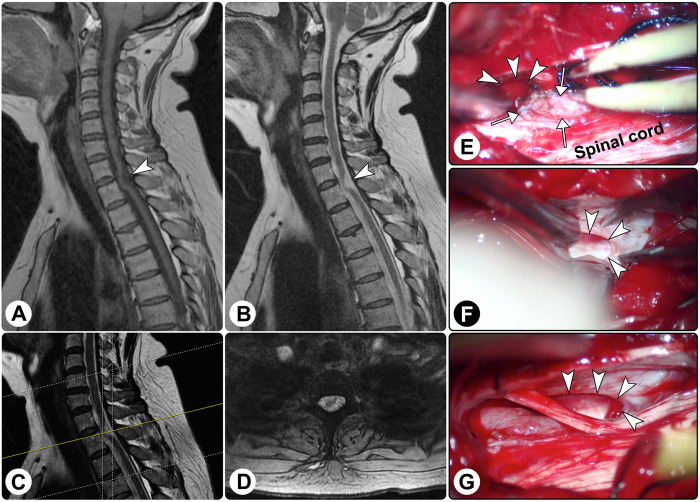
Spinal magnetic resonance imaging and intraoperative findings. Spinal magnetic resonance imaging showed the spinal cord was distorted ((**A**) sagittal T1-weighted imaging; (**B**) sagittal T2-weighted imaging; (**C**) the yellow line indicating the locating plane; (**D**) axial T2-weighted imaging). (**E**–**G**) Intraoperatively, a dural defect was found (white arrowheads), and surgical reduction of the herniated spinal cord (white arrows) was performed.

Spinal magnetic resonance imaging (MRI) demonstrated ventral displacement of the spinal cord at the C7–T1 levels, and there seemed to be a cuneiform space-occupying lesion dorsal to the spinal cord with hypointensity on T1-weighted imaging and hyperintensity on T2-weighted imaging (Figures 1A–D). A diagnosis of the spinal intradural extramedullary space-occupying lesion was suspected.

### Surgical Treatment and Intraoperative Findings

An exploratory operation was performed via a posterior midline approach. Intraoperatively, after the dura matter and arachnoid membrane were opened, no lesion was found except for the arachnoid hyperplasia, and the spinal cord was distorted ventrally. Further exploration revealed a defect in the ventral dura, which was approximately 1 cm × 2 cm in size, and the spinal cord herniated to the epidural space through the defect (Figures 1E–G). After the herniated parenchyma was returned, an artificial dura matter was used to repair the defect. Additionally, the surrounding adhesion was released.

### Follow-up

The postoperative course was uneventful. After a 3-month follow-up, the lower-extremity weakness was significantly improved (muscle strength 5/5), while the sensation disturbance remained unchanged. There was no recurrence of the spinal cord herniation.

## Discussion

According to etiology, spinal cord herniation can be classified into three subtypes: idiopathic, post-traumatic, and iatrogenic (13). Idiopathic spinal cord herniation is also known as “idiopathic ventral spinal cord herniation” or “idiopathic thoracic spinal cord herniation” since the defect is exclusively located in the ventral dura and this disorder only affects the thoracic region. There are several theories regarding the mechanisms of the dural defect formation and the secondary spinal cord herniation:

1) The “occult injuries” theory. Some scholars postulated that the dural defect might be attributed to repetitive trivial injuries in activities involving excessive extension and rotation of the spine (14, 15). These occult injuries may cause micro-damage to the dura mater unconsciously, and after a long period of time, the accumulated damage forms a pathological basis of the dural defect.
2) The “pressure erosion” theory. Isu et al. proposed that the pressure erosion attributable to intradural arachnoid cysts may be the cause of the dural tear (16). However, in the majority of previously reported cases, as well as in our case, no arachnoid cyst was found during the operation, and there was only enlarged cerebrospinal fluid space dorsal to the herniated spinal cord.
3) The “intrathecally ruptured thoracic disc” theory. Wortzman et al. first suggested that the ventral dural defects may be due to intrathecal rupture of disc material; this hypothesis was based on the reports of cases with sequestrated disc embedded in the anterior surface of the spinal cord and on that herniated disc material could induce extensive dural erosion dorsally (1). Subsequently, Massicotte et al. also postulated that the disc might cause the thinning, dehiscence, and even rupture of the overlying dura creating a defect, and herniation of the spinal cord could be induced by cerebrospinal fluid pulsations (17). However, no disc disorder was observed in our case.
4) The “local inflammation” theory. Najjar and colleagues hypothesize that an inflammatory process involving the spinal cord and/or the meninges is the initial event that leads to ventral adhesion of the spinal cord to the dura. The persistent inflammation leads to dural erosion and the formation of a dural defect, and as a result, the spinal cord is tethered and later starts to herniate through the dural defect with cerebral spinal fluid pulsations (18). In our patient, extensive adhesions were encountered at the dural edge of the dural defect, which prevented the reduction of the herniated spinal cord.
5) The most popular theory is “congenital defects or dural dysplasia”. Some scholars proposed that the spinal cord may herniate into a preexisting ventral meningocele (1, 19). However, meningoceles are usually situated laterally and associated with local dural dysplasia or preexisting skeletal defects. Recently, Bartels and coworkers performed a neuropathological analysis on a patient with idiopathic spinal cord herniation, and they proposed a new embryologic explanation (4). At the gestational age of 30–60 days, the layer at the ventral side of the neural tube and dorsal side of the intervertebral disk is divided into three sublayers: (a) an outer perichondral sublayer adjacent to the vertebral body, (b) an intermediate sublayer that represents the precursor of the posterior longitudinal ligament, and (c) an internal sublayer forming the ventral dura mater. The authors hypothesized that neural crest cells accumulate and differentiate into neural tissue instead of dura within the internal sublayer, and the aggregate of non-functioning neuronal cells adjoining the spinal cord causes a dural defect (4). Interestingly, Regensburger et al. reported a case of idiopathic spinal cord herniation with long-term longitudinal observation. Neurological examination and spinal MRI were normal during the first clinical visit, while after a symptom-free interval of eight years, MRI showed subtle ventral displacement and posterior indentation of the thoracic spinal cord; After another three years, symptoms were exacerbated, and MRI revealed a severe spinal cord herniation (20). This case seems to argue against the “congenital” theory.

Clinically, idiopathic spinal cord herniation manifests as slow and progressive thoracic myelopathy as it results in spinal cord tethering. Approximately two-thirds of reported cases presented with Brown-Séquard syndrome, and one-third with symmetrical spastic paraparesis. Isolated sensory deficits and sphincter dysfunctions are less common (12). In the current case, the patient presented to us with spastic paraparesis. In recent years, with the advances in MRI techniques, the detection rate of this disease has been significantly improved. According to the rough estimation, the incidence of idiopathic spinal cord herniation may account for 0.059%–0.08% of cases necessitating spinal surgery (21, 22). Currently, this disease is overlooked, and the actual affected cases are greatly underestimated. On sagittal MRI, idiopathic spinal cord herniation shows the ventral angulation of the thoracic spinal cord and enlarged subarachnoid space in the dorsal region, forming a “delta” configuration. On axial MRI, some authors noted the ventral herniation of the spinal cord (4, 23); nevertheless, no specific appearance was noted in our case, leading to diagnostic challenges. Noteworthily, in the vast majority of reported cases, idiopathic spinal cord herniation affects the midthoracic region, and that occurring at the T1 level has only been reported in one case (23). Herein, we reported a case of idiopathic spinal cord herniation occurring at the C7–T1 levels, which adds clinical evidence to the literature. Differential diagnoses of idiopathic spinal cord herniation mainly include dorsal arachnoid cyst, astrocytoma, disc herniation, extradural compression, and transverse myelitis (12).

Currently, no standardized regimen has been established for the treatment of idiopathic spinal cord herniation. Intraoperative ultrasound is a useful tool to identify the location of the herniation before performing a durotomy (24, 25). In the early years, direct closure of the dural defect via the anterior approach was attempted (14). Recently, the posterior approach is more popularly used, and the main goal of surgery is to release the spinal cord tethering and return the spinal cord to the normal anatomical position; the necessity of defect repair remains controversial (26). In addition, Najjar et al. suggested that widening the dural defect may be superior to the grafting of the defect (18). In the current case, we used artificial dura matter to repair the dural defect, leading to a satisfactory prognosis.

We searched PubMed, Embase, and Web of Science (up to and including April 2022) for published articles using the search terms “spinal cord herniation” and a total of 251 patients with idiopathic spinal cord herniation were identified. Individual patient information was collected, and a pooled analysis was performed incorporating the case data in the current study. There were 156 females and 96 males, yielding a female-to-male ratio of 1.63:1. The average age was 51.6 ± 12.1 years (range, 20–78 years), and the mean duration of symptoms before surgery was 4.2 ± 4.5 years (range, 0.1––32 years). Onset symptoms included Brown-Séquard syndrome (58.6%; *n* = 139/237), myelopathy (31.2%; *n* = 74/237), spastic paraparesis (13.9%; *n* = 33/237), and radiculopathy (1.3%; *n* = 3/237). Mid-thoracic segments (T5–T8; 66.0%) were the predilection site, followed by upper-thoracic segments (T1–T4; 30.0%) and lower-thoracic segments (T9–T12; 4.0%); no idiopathic spinal cord herniation occurring at cervical or lumbar levels was reported. A total of 136 patients were treated with duraplasty using graft or patch, 56 patients were treated with defect widening, and 17 patients were treated with primary suture closure. Conservative observation was adopted in 16 cases. Postoperatively, the symptoms were improved in 175 (72.9%) patients and remained unchanged in 48 (20.0%) patients; 17 (7.1%) patients experienced neurological deterioration.

## Conclusion

We reported an extremely rare case of idiopathic spinal cord herniation occurring at the C7–T1 levels. Surgical reduction of the herniated spinal cord with the repair of the dural defect is an effective approach for treatment, and the surgical outcome is satisfactory.

## Data Availability

The raw data supporting the conclusions of this article will be made available by the authors, without undue reservation.
